# Pediatric Case of Severe COVID-19 With Shock and Multisystem Inflammation

**DOI:** 10.7759/cureus.8915

**Published:** 2020-06-29

**Authors:** David C Nguyen, Hanan Haydar, Elizabeth R Pace, Xiaochun Susan Zhang, Katherine R Dobbs

**Affiliations:** 1 Pediatric Infectious Diseases, University Hospitals Rainbow Babies and Children's Hospital, Cleveland, USA; 2 Pediatric Critical Care Medicine, University Hospitals Rainbow Babies and Children's Hospital, Cleveland, USA; 3 Pathology, University Hospitals Cleveland Medical Center, Cleveland, USA; 4 Pediatrics, Case Western Reserve University School of Medicine, Cleveland, USA

**Keywords:** covid-19, mis-c, sars-cov-2, pediatric, multisystem, inflammation

## Abstract

While the majority of pediatric coronavirus disease 2019 (COVID-19) cases have not been critical, occurrences of a multisystem inflammatory syndrome in children (MIS-C) have been emerging as the pandemic progresses. Herein, we report our experience with a pediatric COVID-19 case that presented with shock and multisystem inflammation. Our patient notably had multiple negative severe acute respiratory syndrome coronavirus 2 (SARS-CoV-2) reverse transcription polymerase chain reaction (RT-PCR) assays but tested positive for SARS-CoV-2 IgG antibody. This case not only highlights the utility of SARS-CoV-2 IgG in the diagnosis of COVID-19 when RT-PCR is negative but suggests MIS-C may be a post-infectious immune-mediated process.

## Introduction

Since December 2019, a novel coronavirus now designated severe acute respiratory syndrome coronavirus 2 (SARS-CoV-2) has rapidly spread, leading to a global pandemic of coronavirus disease 2019 (COVID-19). SARS-CoV-2 has infected people of all ages, but the majority of pediatric cases reported have been asymptomatic, mild, or moderate [1,2]. To date, reverse transcription polymerase chain reaction (RT-PCR) has been the most common method to detect the virus, but sensitivity depends on the timing of testing relative to a patient’s disease course [3,4]. SARS-CoV-2 antibody testing may aid in diagnosis when RT-PCR is negative [3,5]. We describe a pediatric patient presenting in shock with multisystem inflammation with negative SARS-CoV-2 PCR testing and positive SARS-CoV-2 IgG.

## Case presentation

A 10-year-old girl with no significant medical history presented with an eight-day history of fevers, sore throat, abdominal pain, diarrhea, and occasional emesis. Her temperature was reported to have been as high as 40°C at home. She had previously presented to our emergency department on day 3 of her illness. At that time, the patient’s parents had reported that her father had tested positive for SARS-CoV-2 four weeks before the onset of her symptoms. His infection had been mild and he had not been hospitalized. Additionally, the patient’s mother reported her own mild cough and congestion one and a half weeks prior to this presentation but was never tested for SARS-CoV-2. SARS-CoV-2 RT-PCR of the patient’s nasopharyngeal specimen was negative. A complete blood count showed mild lymphopenia with a total lymphocyte count of 1.56 × 10^9^ cells/L. A C-reactive protein (CRP) was mildly elevated to 3.19 mg/dL. Her remaining laboratory tests for that day were unremarkable. She was sent home on supportive management.

The patient returned to our emergency department on day 8 of illness due to continued fever, abdominal pain, diarrhea, sore throat, nasal congestion, and poor oral intake with subsequent decreased urine output. Her temperature was 39.1°C, blood pressure 83/45 mmHg, heart rate 137 beats/min, respiratory rate 44 breaths/min, and oxygen saturation 99% in ambient air. On examination, she was ill-appearing but not toxic, her extremities were cool, she was not in respiratory distress and her lungs were clear to auscultation. Scattered faint erythematous annular lesions 1.5 cm in diameter were noted over her chest, right upper back, and arms. Laboratory testing showed a white blood cell count of 13.3 × 10^9^ cells/L, decreased lymphocyte count 0.93 × 10^9^ cells/L, CRP markedly increased to 14.32 mg/dL, erythrocyte sedimentation rate (ESR) mildly elevated at 25 mm/hour, and mild transaminase elevation with alanine transaminase (ALT) of 66 U/L and aspartate transaminase (AST) of 79 U/L. Other laboratory results and trend are shown in Table 1.

**Table 1:** Trend of laboratory values WBC, white blood cell; PT, prothrombin time; INR, international normalized ratio; ALT, alanine transaminase; AST, aspartate transaminase; ESR, erythrocyte sedimentation rate; BNP, B type brain natriuretic peptide.

	Hospital Day 1	Hospital Day 2	Hospital Day 3
WBC (reference range 4.5-14.5 × 10^9 ^cells/L)	9.2	10.3	10.1
Hemoglobin (reference range 11.5-15.5 g/dL)	8.9	8.7	8.9
Platelet (reference range 150-400 × 10^9 ^cells/L)	181	196	239
Lymphocyte count (reference range 1.8-5.0 × 10^9 ^cells/L)	0.93	1.68	2.05
PT (reference range 9.7-12.7 seconds)	14.8	15.1	14.4
INR (reference range 0.9-1.1 seconds)	1.3	1.4	1.3
D-dimer (reference range ≤ 500 ng/mL)	5,299	6,612	3,355
Fibrinogen (reference range 200-400 mg/dL)	450	412	373
Bicarbonate (reference range 18-27 mmol/L)	16	21	25
Creatinine (reference range 0.30-0.70 mg/dL)	0.32	0.25	0.27
Albumin (reference range 3.5-5 g/dL)	2.8	2.6	2.5
ALT (reference range 3-28 U/L)	66	52	40
AST (reference range 13-32 U/L)	70	60	31
Ferritin (reference range 8-150 µg/mL)	259	291	232
C-reactive protein serum (reference range <1 mg/dL)	14.3	11.18	11.88
ESR (reference range 0-13 mm/hour)	25		
BNP (reference range 0-99 pg/mL)	438		376
Troponin (reference range 0-0.03 ng/mL)	0.08	0.08	0.05

**Figure 1:**
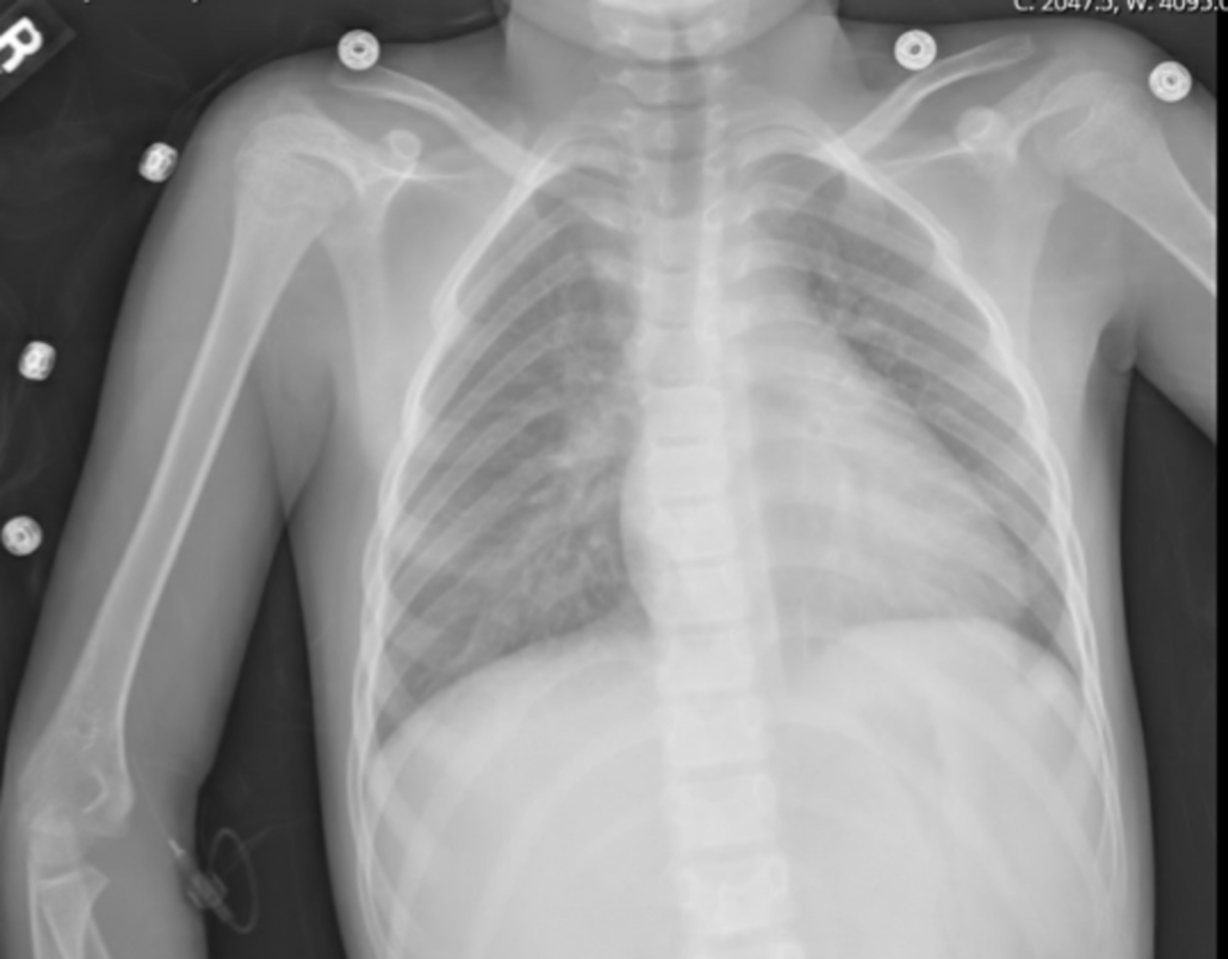
Chest x-ray showing perihilar peribronchial thickening without focal consolidation

A second nasopharyngeal SARS-CoV-2 RT-PCR was negative. A serum heterophile antibody and group A Streptococcus PCR of a throat swab were also negative. A chest x-ray showed perihilar peribronchial thickening without focal consolidation (Figure 1).

She received a total 80 mL/kg 0.9% NaCl solution intravenous fluid boluses and was admitted to the pediatric intensive care unit for fluid-refractory shock. Venous lactate remained non-elevated throughout admission. Blood and urine cultures were collected, and she was empirically started on intravenous vancomycin 15 mg/kg every six hours and ceftriaxone one gram every 24 hours. Other testing included a stool pathogen PCR panel, Epstein-Barr virus serology panel from serum, and nasopharyngeal PCR testing for influenza A and B, respiratory syncytial virus, adenovirus, rhinovirus, human metapneumovirus, and parainfluenza virus, all of which resulted negative.

Despite two negative RT-PCR tests, there remained a high suspicion for COVID-19 given her clinical and laboratory findings and exposure through a positive family member. Additional laboratory testing showed ferritin 291 µg/mL, D-dimer 6,612 ng/mL, fibrinogen 450 mg/dL, and prothrombin time (PT) 14.6 seconds. Hematology was consulted for her coagulopathy and venous thromboembolism (VTE) prophylaxis and recommended enoxaparin 0.5 mg/kg twice daily. She was maintained on only enoxaparin at prophylactic dosing during her hospital stay.

The patient also had abnormal cardiac markers. She had elevated brain natriuretic peptide of 438 pg/mL and elevated troponin I of 0.08 ng/mL. An electrocardiogram showed normal sinus rhythm and no ST segment changes. A transthoracic echocardiogram showed normal ventricular function and no evidence of pericardial effusion or coronary artery dilation. Troponin I was serially monitored and decreased without intervention. 

The patient was afebrile after 24 hours of admission, and her heart rate and blood pressure improved. After her initial fluid resuscitation, no vasoactive, antiviral, or oxygen therapy was required. Abnormal laboratory values improved on serial measurement. One blood culture from the day of admission grew coagulase-negative Staphylococcus, and this was thought to be a likely contaminant. A follow-up blood culture had no growth.

A search for confirmation of COVID-19 continued during her hospitalization. SARS-CoV-2 RT-PCR tests from her stool and a third nasopharyngeal swab both resulted negative. Serum from hospital day 2 and 3 was sent for SARS-CoV-2 IgG testing and resulted positive shortly after the patient had been discharged on hospital day 4. The SARS-CoV-2 IgG assay utilized in this case was a chemiluminescent microparticle immunoassay performed on the Architect i1000SR instrument (Abbott, Chicago, IL). The assay detects IgG antibodies against the nucleocapsid protein of SARS-CoV-2. The SARS-CoV-2 IgG index value on hospital day 2 (nine days post-symptom onset) and day 3 (10 days post-symptom onset) was 6.77 and 6.98, respectively, and assay cutoff for a positive result is 1.4. The results suggested a strong immune response to the virus.

## Discussion

This is a pediatric case of shock and multisystem inflammation temporally associated with COVID-19. Newer studies have demonstrated that COVID-19 can result in a substantial disease burden in the pediatric population, though severe illness is less commonly seen [6]. This patient’s clinical presentation of fever, abdominal pain, diarrhea, sore throat, and ultimately fluid-refractory shock was more severe than has been described for the many pediatric COVID-19 cases [1,2]. Additionally, she had abnormal laboratory findings indicative of hyperinflammation similar to those described in recent case series of pediatric patients afflicted by the newly defined multisystem inflammatory syndrome in children (MIS-C), including lymphocytopenia, elevated CRP, elevated transaminases, and elevated D-dimer [7-9].

We have noted a case report accepted for publication of a six-month-old female with a positive SARS-CoV-2 RT-PCR who presented with classic Kawasaki disease (KD), including a polymorphous maculopapular rash [10]. Health alerts released in the United Kingdom and United States for MIS-C indicate the syndrome has features of toxic shock syndrome and KD and laboratory findings of severe COVID-19; abdominal pain, gastrointestinal symptoms, and cardiac inflammation have been reported [7,9]. While our patient did not meet classic criteria for KD, she did warrant an evaluation for incomplete/atypical KD given her prolonged fever, elevated CRP, elevated ALT, low albumin, and anemia. Initial echocardiogram showed normal coronary arteries, and the patient defervesced on hospital day 2. Repeat echocardiogram at two weeks was recommended. The similar findings between KD and severe COVID-19 likely reflect excessive inflammatory cytokine production that underlies both disease processes.

Overall, this patient’s presentation was more of a systemic illness than a chiefly pneumonic process; as such, we felt it was important to seek diagnostic confirmation for COVID-19 after excluding alternative infectious diagnoses. Currently, there is uncertainty regarding the optimal testing approach for COVID-19 in children. Likewise, case definitions for MIS-C do not recommend one specific site for RT-PCR testing or a specific RT-PCR and serologic testing sequence. Cases of suspected COVID-19 with negative RT-PCR testing have been described in the literature, including in pediatrics [1,5]. Sensitivity of testing by respiratory RT-PCR appears to be lower later in the disease course [3,4]. By contrast, IgM and IgG antibodies against SARS-CoV-2 may be detectable a few days after symptom onset with a dramatic increase in sensitivity after one week [3,5]. This patient’s consistently negative nasopharyngeal RT-PCR tests and positive IgG are consistent with a possible post-infectious immune-mediated process. Interestingly, it has been observed that stool or anal RT-PCR detects SARS-CoV-2 RNA longer than those of nasopharyngeal samples despite some having no gastrointestinal symptoms [4,11]. This patient had a negative stool RT-PCR and did have abdominal pain, emesis, and diarrhea. This seemingly contradictory observation speaks to the need to further study detectable viral RNA in stool and its role in confirming a COVID-19 diagnosis.

Finally, this patient was evaluated for coagulopathy given suspicion for COVID-19 and found to have markedly elevated D-dimer and prolonged PT. Coagulopathy has been observed in some adult COVID-19 patients and has been associated with poorer prognosis [12,13]. The exact cause of coagulopathy in COVID-19 is unknown but may be the effect of systemic inflammation, liver dysfunction, and/or endothelial dysfunction. Coagulopathy along with systemic inflammation and critical illness may predispose patients to thrombotic events. Klok et al. found a 31% incidence of thrombotic events in 184 adult ICU patients with COVID-19 [14]. The incidence of thrombotic events in non-ICU patients, outpatients, and pediatric patients with COVID-19 is not known. Nevertheless, it has been recommended to administer low-molecular-weight heparin for VTE prophylaxis for hospitalized COVID-19 patients [15,16]. None of these recommendations were pediatric specific, but in consultation with our hematology colleagues our patient received prophylactic enoxaparin while she was hospitalized. There is currently no guidance on the need to continue VTE prophylaxis after discharge.

## Conclusions

This case highlights the potential utility of SARS-CoV-2 antibody testing in the diagnosis of COVID-19 when RT-PCR is negative. This may be especially true for patients who present later in their disease course with symptoms and laboratory testing indicative of extensive hyperinflammatory response. Development of optimal diagnostic testing algorithms for COVID-19 is needed and may have to take into account timing of presentation. We also hope this case provides insights into a more severe systemic presentation of COVID-19 in a child with no comorbidities, a clinical scenario earning more recent attention in the pediatric literature surrounding COVID-19. This patient’s presentation may not have represented disease caused by acute infection but rather the recently defined MIS-C following infection in the preceding weeks.
